# Targeting connexin 43 provides anti-inflammatory effects after intracerebral hemorrhage injury by regulating YAP signaling

**DOI:** 10.1186/s12974-020-01978-z

**Published:** 2020-10-28

**Authors:** Hailong Yu, Xiang Cao, Wei Li, Pinyi Liu, Yuanyuan Zhao, Lilong Song, Jian Chen, Beilei Chen, Wenkui Yu, Yun Xu

**Affiliations:** 1 Affiliated of Drum Tower Hospital, Medical school of Nanjing University, Nanjing, Jiangsu People’s Republic of China; 2 Clinical Medical College of Yangzhou University, Yangzhou, Jiangsu People’s Republic of China; 3 Department of Neurology, Northern Jiangsu People’s Hospital, Yangzhou, Jiangsu People’s Republic of China; 4 Dalian Medical University, Dalian, Liaoning People’s Republic of China

**Keywords:** Intracerebral hemorrhage injury, Connexin 43, Hemichannels, Reactive astrocyte, YAP

## Abstract

**Background:**

In the central nervous system (CNS), connexin 43 (Cx43) is mainly expressed in astrocytes and regulates astrocytic network homeostasis. Similar to Cx43 overexpression, abnormal excessive opening of Cx43 hemichannels (Cx43Hcs) on reactive astrocytes aggravates the inflammatory response and cell death in CNS pathologies. However, the role of excessive Cx43Hc opening in intracerebral hemorrhage (ICH) injury is not clear.

**Methods:**

Hemin stimulation in primary cells and collagenase IV injection in C57BL/6J (B6) mice were used as ICH models in vitro and in vivo. After ICH injury, the Cx43 mimetic peptide Gap19 was used for treatment. Ethidium bromide (EtBr) uptake assays were used to measure the opening of Cx43Hcs. Western blotting and immunofluorescence were used to measure protein expression. qRT-PCR and ELISA were used to determine the levels of cytokines. Coimmunoprecipitation (Co-IP) and the Duolink in situ proximity ligation assay (PLA) were applied to measure the association between proteins.

**Results:**

In this study, Cx43 expression upregulation and excessive Cx43Hc opening was observed in mice after ICH injury. Delayed treatment with Gap19 significantly alleviated hematoma volume and neurological deficits after ICH injury. In addition, Gap19 decreased inflammatory cytokine levels in the tissue surrounding the hematoma and decreased reactive astrogliosis after ICH injury in vitro and in vivo. Intriguingly, Cx43 transcriptional activity and expression in astrocytes were significantly increased after hemin stimulation in culture. However, Gap19 treatment downregulated astrocytic Cx43 expression through the ubiquitin-proteasome pathway without affecting Cx43 transcription. Additionally, our data showed that Gap19 increased Yes-associated protein (YAP) nuclear translocation. This subsequently upregulated SOCS1 and SOCS3 expression and then inhibited the TLR4-NFκB and JAK2-STAT3 pathways in hemin-stimulated astrocytes. Finally, the YAP inhibitor, verteporfin (VP), reversed the anti-inflammatory effect of Gap19 in vitro and almost completely blocked its protective effects in vivo after ICH injury.

**Conclusions:**

This study provides new insight into potential treatment strategies for ICH injury involving astroglial Cx43 and Cx43Hcs. Suppression of abnormal astroglial Cx43 expression and Cx43Hc opening by Gap19 has anti-inflammatory and neuroprotective effects after ICH injury.

## Background

Intracerebral hemorrhage (ICH) refers to the spontaneous rupture of blood vessels in the brain and leads to high morbidity, disability, and mortality [1]. The formation of a large hematoma that compresses the surrounding brain tissue results in neurological deterioration, intracranial hypertension, and intracerebral hernia [2]. Various endogenous molecules are released into the brain parenchyma resulting in cytotoxicity and excitotoxicity through neuroinflammation, exacerbation of oxidative stress, and disruption of cell signaling pathways, leading to secondary brain damage during ICH [3]. During ICH injury, astrocytes can be activated by hematoma degradation products, such as hemin chloride. These activated astrocytes accumulate in the area surrounding the hematoma and can produce and release cytokines and chemokines, further aggravating the inflammatory responses. Recent studies have reported that inflammatory responses caused by reactive astrocytes play a key role in blood-brain barrier (BBB) destruction and neuronal apoptosis during ICH [4]. Under physiological and pathological conditions, astrocytes maintain communication with the neural microenvironment through gap junction channels (GJCs). GJCs consist of two juxtaposed half channels (hemichannels, Hcs) between adjacent cells. Hcs are mainly involved in the exchange of ions and small molecules between the intracellular and extracellular environments [5]. Physiologically, Hcs are in the off state. However, under pathological conditions, such as stroke, the excessive opening of Hcs leads to the release of inflammatory factors, glutamate, and ATP [6, 7]. Connexin proteins are the basic unit of Hcs. Eleven types of connexin proteins have been identified thus far in the central nervous system (CNS), and connexin 43 (Cx43) is the main gap junction protein in astrocytes [8]. Previous studies have shown that, like Cx43 overexpression, excessive opening of Cx43 hemichannels (Cx43Hcs) on the surface of reactive astrocytes results in the release of inflammatory factors that aggravate the inflammatory response of the local environment and recruit immune cells to the damaged area under pathological conditions [8, 9]. Our previous studies confirmed that astrocytic Cx43 overexpression and excessive Cx43Hc opening aggravate apoptosis and inflammation after cerebral ischemic injury [10, 11]. These pathological processes are reversed by Cx43 mimetic peptides, including Gap26, Gap27, and Gap19, which simulated the short nucleic acid sequences derived from the loop of Cx43. They possess the ability to selectively block Cx43Hcs in the CNS [12]. Moreover, Gap19 exerts more protective effects after cerebral ischemic injury than Gap26 or Gap27 [10, 13]. The reason for this may be that Gap19 is more selective in inhibiting Cx43Hcs compared to Gap26 and Gap27 without influencing Cx43GJCs, which are beneficial in CNS diseases [12]. Intriguingly, linking a TAT membrane translocation motif to the Gap19 N-terminus significantly increases the ability of Gap19 to cross the BBB [12]. Therefore, we employed Gap19 to block Cx43Hc opening in this study.

Yes-associated protein (YAP) is the transcriptional coactivator of the Hippo signaling pathway. Inactivation of Hippo signaling enhances the dephosphorylation state of YAP, which improves its nuclear capability, leading to antiproliferative and apoptotic effects under different pathophysiological conditions [14]. Unlike other core proteins of the Hippo signaling pathway (MSTs, LATS, and TAZ), YAP has been suggested to be strictly located in astrocytes and neural stem cells. Specifically, members of the TEAD family, including TEAD1, TEAD2, and TEAD3, are the core transcription factors of YAP in the nucleus and are expressed in astrocytes [15]. YAP, the transcriptional coactivator of Hippo signaling, has largely been studied for its role in the immune response and as a potential transcriptional regulation of the tumor checkpoint receptor PD-1 [16, 17]. Recently, YAP was investigated for its ability to inhibit the overactivation of astrocytes, which attenuates inflammation in the CNS. Interestingly, YAP has also been found to be sequestered at cell junctions and mechanically bound to Cx43 [18]. In this study, we show that the Cx43 mimetic peptide Gap19 inhibits both Cx43 expression at the protein level and excessive opening of Cx43Hcs on astrocytes in mice after ICH. Furthermore, we show that Gap19 provides neuroprotection and promotes cell survival by inhibiting inflammation-associated regulation of YAP signaling in reactive astrocytes after ICH injury.

## Methods

### Animals

All adult male C57BL/6J (B6) mice (8–10 weeks, 25–30 g) were purchased from the Animal Model Center of Nanjing University. All experimental B6 mice were maintained in an air-conditioned room at 25 °C. The animals were reared on a 12 h light/dark cycle and provided sufficient food and water. All experimental procedures were approved by the Animal Ethics Committee of Nanjing University and conducted under the guidance of the National Institutes of Health Laboratory Animal Care and Use Guidelines and the Nanjing University Laboratory Animal Management Regulations. All efforts were made to reduce pain in the animals and to obtain as many results from as few mice as possible.

### Mice ICH model and treatment administration

Mice were anesthetized with an isoflurane-oxygen mixture during the surgical procedure, and the animal’s body temperature was maintained at 37.0 ± 0.5 °C using a heating blanket. Mice were then placed in an astereotaxic frame, and a coronal incision was performed to expose the cranium until the bregma was clearly visible. A hole was drilled 0.5 mm posterior to bregma and 2 mm lateral to the left (striatal part). The depth of the microsyringe needle was adjusted to 3.5 mm. A microinfusion pump was connected to a 10 μL syringe, and 0.6 μL of saline containing 0.075 U collagenase IV (Sigma-Aldrich, MO, USA) was injected into the striatum at a rate of 0.12 μL/min. After the injection was completed, the syringe was left in place for 5 min and slowly withdrawn at a rate of 1 mm every 3 min to prevent backflow after the injection. The drill hole was then sealed with bone wax, and overlying skin incision sutured. The model mice were then allowed to recover on a heating pad at 37 °C, and the neurological deficits were closely observed.

TAT-Gap19 (25 mg/kg, Tocris, #6227) was first administered via intraperitoneal injection (i.p.) 1 h after ICH and then administered daily for 2 consecutive days. The YAP inhibitor verteporfin (Selleck, #1786) was injected into the lateral ventricle (0.1 mg/kg, injected slowly over 10 min) at the same time points via TAT-Gap19.

### Behavioral analysis

On the third day after ICH induction, the neurological deficits in each mice were quantified using the modified neurological severity score (mNSS), balance beam test, forelimb strength test, and foot failure test [10]. All procedures were performed by two independent investigators blinded to the experimental groups.

#### mNSS score

This neurological function score ranges from 0 to 18 with a normal score being 0 and a higher score reflecting more serious neurologic deficit.

#### Balance beam experiment

Three days before ICH modeling, mice were trained on balance beam walking skills. The test was then completed on the third day after ICH induction. The mouse is then placed on the balance beam and the gait pattern scored on a score of 0 to 6.

#### Determination of forelimb strength

Mouse forelimb strength test was conducted to detect the recovery of limb function. After the grip meter is zeroed, the mouse is placed on the platform in front of the grip rod. Animals instinctively grab any object when moving backwards unintentionally to prevent backwards until the pulling force exceeds their grip. The tail of the mouse is pulled such that the mouse grasps the grip rod. After the animal loses its grip, the tester automatically records the maximum tensile force. From three consecutive measurements, the instrument automatically records the maximum grip strength recorded.

#### Foot fault test

The foot fault test (FFT) assessed the ability of mice to place their paws on the grid. The mice were trained 3 days before the operation, and each training session lasted for 5 min for at least twice a day. At the end of the third day of training, the average performance of each mouse was taken as the baseline. On the third day after ICH, the mice were placed on a horizontal metal grid. The total number of steps taken and the number of times the left forelimbs slipped off the grid within 2 min was recorded.

### Cultivation of primary astrocytes and treatment administration

Primary astrocytes were obtained from suckling mice within 24 h after birth. Gray cortices were minced and digested. After centrifugation, precipitates were resuspended in DMEM medium with 10% FBS and 100 U/mL penicillin, 100 mg/mL streptomycin.

The astrocytes were cultured in different environments: (1) the control group, cultured with an equal volume of medium; (2) the Gap19 group, cultured with Gap19 (100 μM/mL, using the same dosage described above) in an equal volume of culture medium; (3) the VP group, cultured with VP (5 μM/mL, using the same dose mentioned above) to the same volume of medium; and (4) the hemin group, cultured with hemin (20 μM/mL, using the same dose mentioned above) to the same volume of medium. Gap19 and VP were added to the medium at the beginning of hemin stimulation. The cells were used for protein, RNA extraction, or immunofluorescence staining after 12 h of hemin stimulation.

### Cultivation of primary microglia and neurons

Primary microglia are obtained from the cerebral cortex of suckling mice born 1–2 days old. After centrifugation, the precipitates were in DMEM medium with 10% FBS and 100 U/mL penicillin, 100 mg/mL streptomycin. After culturing for 2 weeks, flasks were shaken gently and supernatant containing microglial cells was collected. The primary microglia cells were seeded in 24-well plates (5 × 10^5^ cells/well), cultured in conditioned medium for 24 h, and then conducted subsequent immunofluorescence staining. The primary neurons were obtained from E16–17 mouse embryos. Cells were cultured in Neurobasal media containing 4.5 g/L glucose, supplemented with Glutamax and 2% B27 (Invitrogen, Carlsbad, CA, USA). The primary neurons were seeded in 24-well plates (4 × 10^5^ cells/well), cultured in conditioned medium for 8 h, and then conducted subsequent immunofluorescence staining.

### Lentivirus infection

A lentiviral vector overexpressing Cx43 (Lv-Cx43) and a negative control vector (Lv-con) were purchased from Genechem (Shanghai, China). Astrocytes were cultured and incubated with the lentiviruses. After 24 h, the fresh medium was replaced to remove the excessive transfection complex. The cells from each experimental condition were harvested after 72 h of subculture and used for subsequent experiments.

### Ethidium bromide uptake experiment

Ethidium bromide (EtBr) staining was used to observe the activity of Cx43 hemichannels and the role of Gap19 [11, 19]. For the in vivo studies, tissue sectioning was performed immediately prior to staining as described in Giaume Christian et al. [20]. Brain tissue was harvested and placed in ice-cold artificial CSF (ACSF) containing the following (in mM): 125 NaCl, 2.5 KCl, 25 glucose, 25 NaHCO_3_, 1.25 NaH_2_PO_4_, 2 CaCl_2_, and 1 MgCl_2_ bubbled with 95% O_2_/5% CO_2_, pH 7.4. Brain tissue was sectioned using a vibratome (VT 1000GS; Leica) filled with ice-cold ACSF. The slices were transferred to a holding chamber at room temperature (20–22 °C) and immersed in oxygenated ACSF, pH 7.4, for a stabilization period of 1 h before being used. Acute slices were washed with distilled water and hydrated in PBS and then incubated with a solution containing 0.01% EtBr for 2 min, followed by washing with PBS three times for 10 min each. The sections were then fixed with 4% PFA, washed again, and observed using a fluorescence microscope. ImageJ software was used to analyze the number of EtBr-positive cells in each field of view. For the in vitro experiments, astrocytes, microglia, and neurons were inoculated in 24-well plates (1.5 × 10^5^ cells/well) and then stimulated with hemin and/or Gap19 on glass slides. The cells were then washed twice with PBS and incubated with 0.01% EtBr solution for 5 min. The cells were washed again with PBS and fixed with 4% PFA. The cells were observed and analyzed using the method described above for in vivo staining.

### Luxol fast blue

Luxol fast blue (LFB) staining was used to evaluate the hematoma volumes and white matter damage on the third day after ICH induction. Briefly, 0.1% LFB solution was prepared at 60 °C for 2 h or 37 °C overnight at a constant temperature in an oven. Meanwhile, the prepared frozen sections were dried in an oven at 37 °C for 1 h. The sections were hydrated in a 95% ethanol solution for 1 min, placed in the preheated 0.1% LFB solution for 2 h, and then cooled for 30 min. The sections were rinsed in 0.03% lithium carbonate and 70% ethanol for 2 min each. The above steps were repeated until the difference between the gray matter and white matter was obvious. Then, the sections were dehydrated in an ethanol solution for 2 min and then sealed with transparent xylene and neutral gum. Damage to the myelin sheath of the white matter after cerebral hemorrhage was analyzed by fluorescence microscopy and ImageJ software.

### Fluoro-Jade C staining

Prepared frozen sections were dried at room temperature (RT), hydrated with distilled water for 2 min, immersed in an 80% ethanol solution containing 1% NaOH for 5 min, and then washed in a 70% ethanol solution, followed by distilled water for 2 min each. The sections were then incubated in 0.06% potassium permanganate for 10 min, washed, and reacted in a 0.0001% fluoro jade C prepared with 0.1% acetic acid working solution for 10 min. After the sections were washed again, they were dried and then sealed with transparent xylene and neutral gum. The apoptotic neurons in the brain tissue around the hemorrhage were observed under a fluorescence microscope.

### Immunofluorescence

The method described above was used to prepare frozen sections on the third day after ICH in model mice or inoculated astrocytes, microglia, and neurons (which were seeded into the 24-well culture plate at 1.5 × 10^5^ cells/well) for immunofluorescence staining [4]. After treatment, tissue sections and cells were incubated overnight with antibodies against GFAP (1:200, Cell Signaling Technology, #80788), Iba1 (1:100, Cell Signaling Technology, #17198), MAP-2 (1:50, SANTA CRUZ, sc-74421), or YAP (1:100, Cell Signaling Technology, #14074). After washing, both were incubated for 2 h with the indicated secondary antibodies (Invitrogen, 1:500) in the dark at RT. After washing in PBS, the samples were stained with 4′,6-diamino-2-phenylindole (DAPI). All staining results were observed using a fluorescence microscope.

### Nuclear/cytoplasmic protein separation and extraction

Nuclear and cytosolic proteins were extracted using a nucleocytoplasmic separation and extraction kit (Thermo-Fisher Scientific, MA, USA) as previously described [21]. The supernatants were collected, and the protein concentrations were measured using the BCA method (Thermo-Fisher).

### Co-immunoprecipitation

After extraction and treatment, different groups of astrocyte proteins were subjected to a co-immunoprecipitation assay. Briefly, astrocytes were inoculated into 6-well plates at a density of 6 × 10^5^, cultured for 24 h under suitable conditions, treated for 24 h with different experimental conditions, and then split and homogenized in immunoprecipitation lysis buffer. The immunoprecipitation samples were collected and incubated with anti-YAP (1:50, Cell Signaling Technology, #14074), anti-Cx43 (1:30, Abcam, #235585), or rabbit IgG (1:20, Cell Signaling Technology, #3424) antibodies at 4 °C with gentle shaking overnight for co-immunoprecipitation. Then, 10 μL of protein A agarose beads were added to the samples, followed by incubation in a 4 °C shaker for an additional 2 h. After centrifugation, the supernatant was carefully removed by suction, and the agarose beads containing the immune complexes were washed 3 times with lysis buffer solution. Finally, sample buffer solution was added, and the samples were boiled for 5 min; the supernatant was collected for subsequent Western blot (WB) analysis.

To demonstrate the interaction between Gap19 and Cx43 or YAP, streptavidin magnetic beads (MCE, HY-K0208) were used. Primary astrocytes were preincubated with biotinylated Gap19 (Gap19-biotin, 100 μM/mL) for 30 min at 4 °C. The other procedures were the same as described above.

### Duolink in situ proximity ligation assay

Proximity ligation assay (PLA) was performed according to the manufacturer’s instructions (Duo92012; Sigma-Aldrich, St. Louis, MO, USA). Briefly, astrocytes were seeded on glass slides, followed by treatment with the above described experimental conditions. The astrocytes were washed, blocked, and then incubated with anti-YAP (1:100) or anti-Cx43 (1:100) primary antibodies overnight at 4 °C. After washing, the astrocytes were incubated with Duolink PLA probes MINUS and PLUS for 1 h at 37 °C. A Duolink in situ detection kit was used for ligation and amplification. DAPI was used to stain the nuclei. The images were captured using a confocal fluorescence scanning microscope, and the red spots indicate the interaction between YAP and Cx43.

### Western blot

At 6 h, 12 h, 24 h, 72 h, and 96 h after ICH, mice were sacrificed, and the brains were quickly removed. The brain tissue around the hemorrhage area was collected, or different astrocytes were collected after different treatments, as described above. The total protein was extracted using a protein extraction kit, and then the protein concentration in the supernatant was measured by the BCA method. After quantitative analysis, equal amounts of proteins were separated by 10% SDS-PAGE and then transferred to PVDF membranes. After blocking with 5% skim milk at RT for 2 h, the PVDF membranes were incubated with the following primary antibodies: anti-IL-1β (1:1000, Cell Signaling Technology, #12703), anti-IL-4 (1:1000, Cell Signaling Technology, #12227), anti-IL-6 (1:1000, Cell Signaling Technology, #12912), anti-IL-10 (1:1000, Cell Signaling Technology, #12163), anti-MCP-1 (1:1000, Cell Signaling Technology, #2027), anti-TNF-α (1:1000, Cell Signaling Technology, #11948), anti-Cx43 (1:1000, Cell Signaling Technology, #3512), anti-YAP (1:1000, Cell Signaling Technology, #14074), anti-phospho-YAP (1:1000, Cell Signaling Technology, #13008), anti-SOCS1 (1:1000, Cell Signaling Technology, #3950), anti-SOCS3 (1:1000, Cell Signaling Technology, #52113), anti-JAK2 (1:1000, Cell Signaling Technology, #3230), anti-phospho-JAK2 (1:1000, Cell Signaling Technology, #3771), anti-STAT3 (1:1000, Cell Signaling Technology, #9139), anti-phospho-STAT3 (1:1000, Cell Signaling Technology, #9145), anti-TLR4 (1:1000, Cell Signaling Technology, #14358), anti-p65 (1:1000, Cell Signaling Technology, #8242), anti-phospho-p65 (1:1000, Cell Signaling Technology, #3033), anti-IKBα (1:1000, Abcam, #32518), anti-IKKβ (1:1000, Cell Signaling Technology, #8943), anti-phospho-IKKβ (1:1000, Cell Signaling Technology, #2697), anti-H3 (1:1000, Cell Signaling Technology, #4499), and anti-tubulin (1:1000, Cell Signaling Technology, #2128), overnight at 4 °C on a shaker. After washing the next day, the membranes were incubated with secondary antibody for 2 h. Subsequently, the enhanced chemiluminescence system (ECL) was used to observe the proteins. The different protein bands were analyzed quantitatively by ImageJ software.

### Total RNA extraction and quantitative real-time PCR analysis

Astrocytes were inoculated and cultured for 24 h under suitable conditions and then treated for the indicated times and experimental conditions as described above. Total RNA was extracted with TRIzol reagent (Invitrogen, CA, USA) and reverse transcribed to cDNA with the PrimeScript RT Reagent Kit (Takara). The expression of IL-1β, IL-6, TNF-α, MCP1, and Cx43 was detected using the SYBR Green Kit (Takara) on an ABI 7500 PCR system. The corresponding primers used were as follows.

### Statistical analysis of the data

All data are expressed as the mean ± SEM of at least three independent experiments. *T* tests were used to compare the means of two groups. One-way ANOVA was used for the comparison of multiple groups. *P* < 0.05 was considered statistically significant, and *P* < 0.01 was considered highly statistically significant. The immunofluorescence images were obtained by a fluorescence microscope and analyzed by ImageJ software. GraphPad Prism 7 (CA, USA) was used to draw the statistical charts.

## Results

### Cx43 overexpression is accompanied by abnormal Cx43Hc opening after ICH injury

**Fig. 1:**
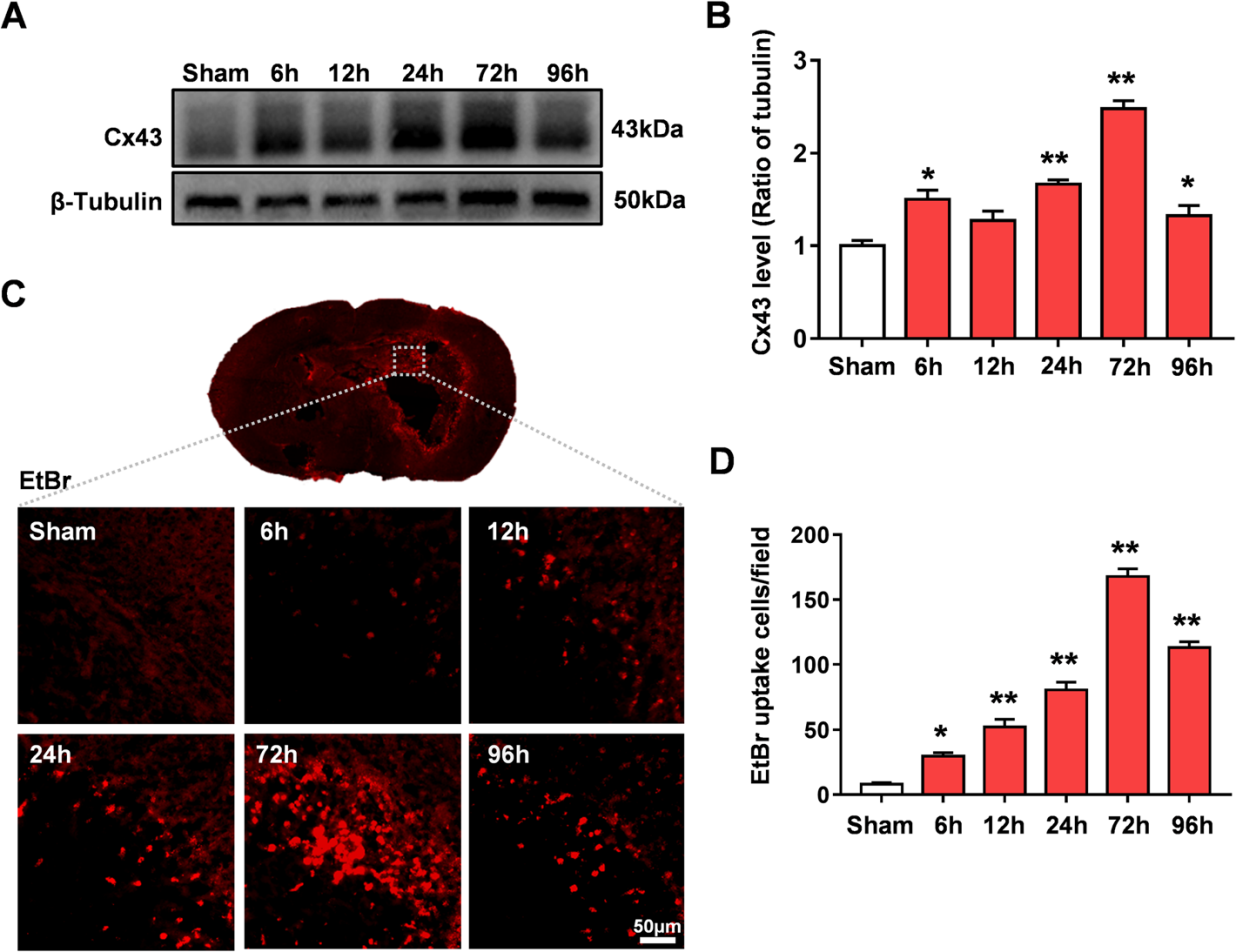
Expression of Cx43 at different time points and abnormal opening of Cx43Hcs after ICH injury. **a** WB analysis of the expression levels of Cx43 and β-tubulin in normal brain tissue and in brain tissue surrounding cerebral hemorrhage 6 h, 12 h, 24 h, 72 h, and 7 days after ICH. **b** Quantitative analysis showing the ratio of Cx43 to β-tubulin. **c** EtBr staining was used to evaluate the opening of the Hcs and to select peripheral brain tissues, as shown in the figure. **d** EtBr staining showed that Hc opening gradually increased after ICH injury, reached a peak at 72 h (excessive Cx43Hc opening), and then gradually decreased at 96 h. The bars represent the SEM of the data from 3 brain samples per group. *P < 0.05, **P < 0.01 compared to the sham group

At present, there is no definitive conclusion on the expression of Cx43 after ICH injury. In this study, Cx43 expression was measured at different time points after ICH. Cx43 expression was upregulated in the ICH group compared to the sham group beginning 6 h after ICH injury, peaked at 72 h after ICH injury and became retuned at 96 h after ICH injury (*P* < 0.05, Fig. 1a, b). Previous studies have revealed that under pathological conditions, Cx43 overexpression is accompanied by abnormal excessive opening of Cx43Hcs [22]. EtBr analysis was employed to detect the opening of Cx43Hcs in our study. The activity of Hcs was significantly increased in the ICH groups compared with the sham group. Interestingly, consistent with the protein level of Cx43, the opening of Hcs gradually increased and peaked 72 h after ICH injury (*P* < 0.01, Fig. 1c, d). These results indicate that Cx43 and Cx43Hcs may play important roles in the pathological process after ICH injury. According to the above results, we chose 72 h after ICH as the observation time point for subsequent studies.

### The Cx43 mimetic peptide Gap19 provides neuroprotection after ICH injury

**Fig. 2:**
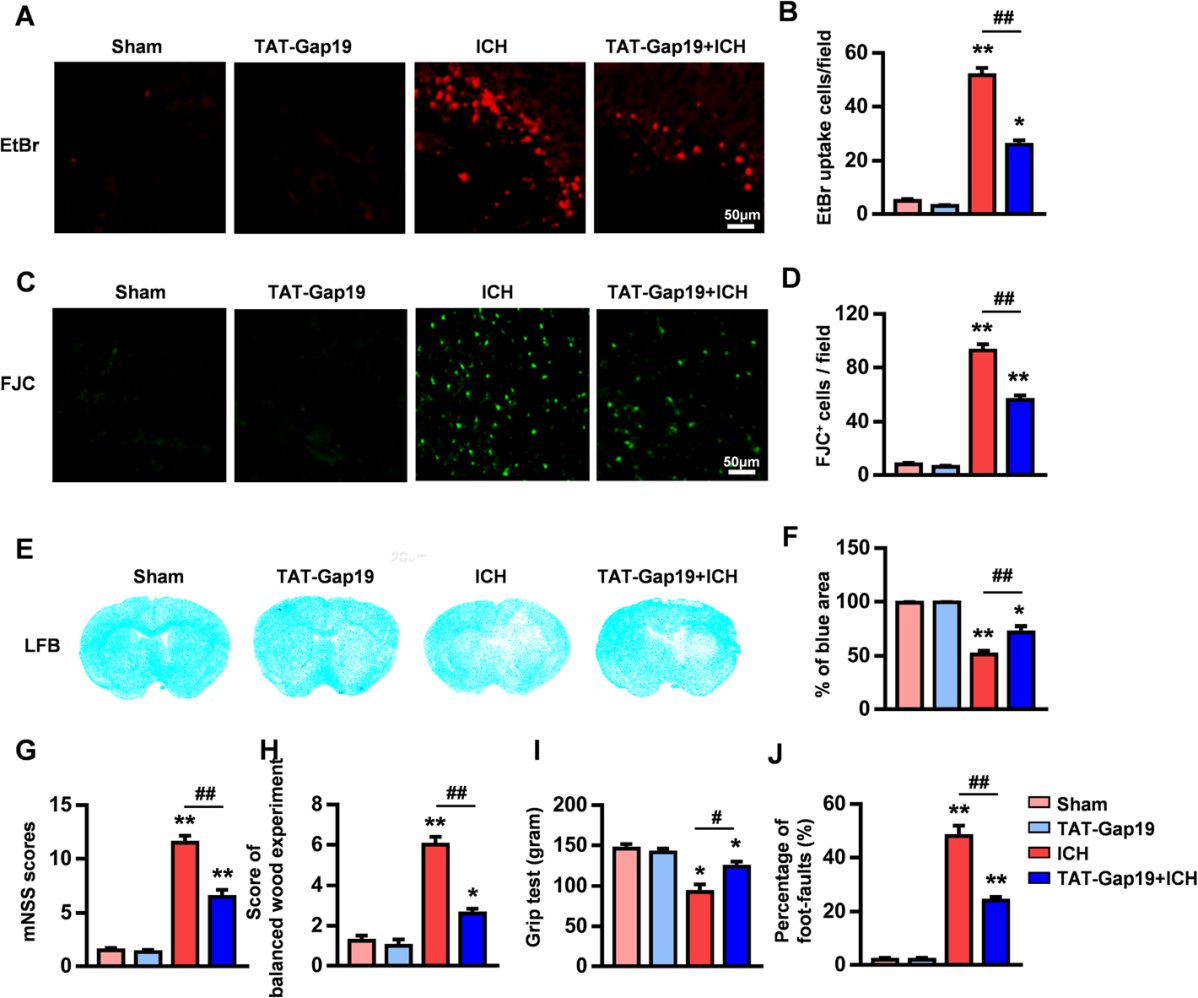
Gap19 blocks Cx43Hc opening and provides neuroprotection after ICH injury. **a**, **b** EtBr staining evaluation showed that TAT-Gap19 treatment after ICH reduced Hc opening. **c**, **d** The TAT-Gap19 ICH group exhibited significantly fewer apoptotic neurons than the ICH group. **e**, **f** TAT-Gap19 treatment reduced hematoma volumes after ICH and increased the surviving blue-stained area. **g** TAT-Gap19 treatment significantly reduced post-ICH neurological dysfunction in mice, as assessed by mNSSs. **h** The balance beam test showed that after TAT-Gap19 treatment following ICH, the balance beam score, which reflected nerve function, was significantly reduced compared with that in the ICH group. **i** In the forelimb strength test, the grip strength of the ICH group was significantly lower than that of the TAT-Gap19 + ICH group. **j** In the foot fault test, the foot fault rate of the TAT-Gap19 + ICH group mice was significantly lower than that of the ICH group. The bars represent the SEM of the data from 3–6 brain tissue samples per group. *P < 0.05, **P < 0.01 compared to the sham group. #*P* < 0.05, ##*P* < 0.01 compared with the ICH group

The Cx43 mimetic peptide Gap19 is used as a selective blocker of Cx43Hcs in the CNS under pathological conditions [23, 24]. Our previous research revealed that Gap19 provides protective effects against cerebral ischemic stroke by reducing infarct volume and alleviating neurological deficits [10, 25]. In this study, we examined its role in the pathological process of ICH injury. The excessive opening of Cx43Hcs was significantly reduced in the TAT-Gap19-treated ICH group compared to the ICH group 72 h after ICH injury (*P* < 0.01, Fig. 2a, b).

Concurrently, the number of apoptotic neurons was also significantly decreased in the TAT-Gap19-treated ICH group compared to the ICH group (*P* < 0.05, Fig. 2c, d). LFB staining can be used to detect white matter damage and to evaluate the hematoma volume after ICH. We stained mouse brain sections and measured the surviving blue-stained area 72 h after ICH. The results showed that, compared to vehicle treatment, TAT-Gap19 treatment significantly reduced the hematoma volume. The blue-stained area of mouse brain sections from the TAT-Gap19 + ICH group was much larger than that of mouse brain sections from the ICH group (71.72 ± 5.719 vs. 51.2 ± 3.507%, *P* < 0.01, Fig. 2e, f).

### Gap19 improves neurological dysfunction after ICH injury

Considering the obvious protective effects of Gap19 treatment in reducing hematoma volume and apoptosis after ICH injury, we further investigated the effects of Gap19 on neural function. Three days after continuous intraperitoneal injection of TAT-Gap19, behavioral tests including the mNSS test, balance beam experiment, forelimb strength test, and foot fault test were performed.

Our data showed that TAT-Gap19 treatment did not influence mouse neurobehavioral function under physiological conditions (*P* > 0.05, Fig. 2g–j), which is consistent with our previous studies [10, 25]. The mNSSs of the mice were significantly increased by ICH damage, while TAT-Gap19 treatment markedly reduced mNSSs (11.20 ± 1.30 vs. 6.50 ± 1.29, *P* < 0.01, Fig. 2g). The balance beam experiment reflects muscle strength and extremity balance. The results showed that the scores of the TAT-Gap19 + ICH group were significantly lower than those of the ICH group (2.6 ± 0.55 vs. 6.00 ± 0.82, *P* < 0.01, Fig. 2h). The grip strength of the ICH group (92.58 ± 9.497 g) was significantly lower than that of the TAT-Gap19 + ICH group (123.6 ± 6.328 g, *P* < 0.05, Fig. 2i). Finally, we detected the foot fault rates of the mice after ICH to evaluate the ability of the mice to integrate and coordinate movement. Compared to the sham group, the foot fault rate of the ICH group was significantly increased, and the TAT-Gap19 + ICH group exhibited a significantly lower foot fault rate than the ICH group (48.00 ± 9.80% vs. 24.00 ± 3.58%, *P* < 0.01, Fig. 2j). These findings confirm that Gap19 exerts a neuroprotective effect in mice after ICH injury.

### Gap19 reduces proinflammatory cytokines in vivo after ICH injury

**Fig. 3:**
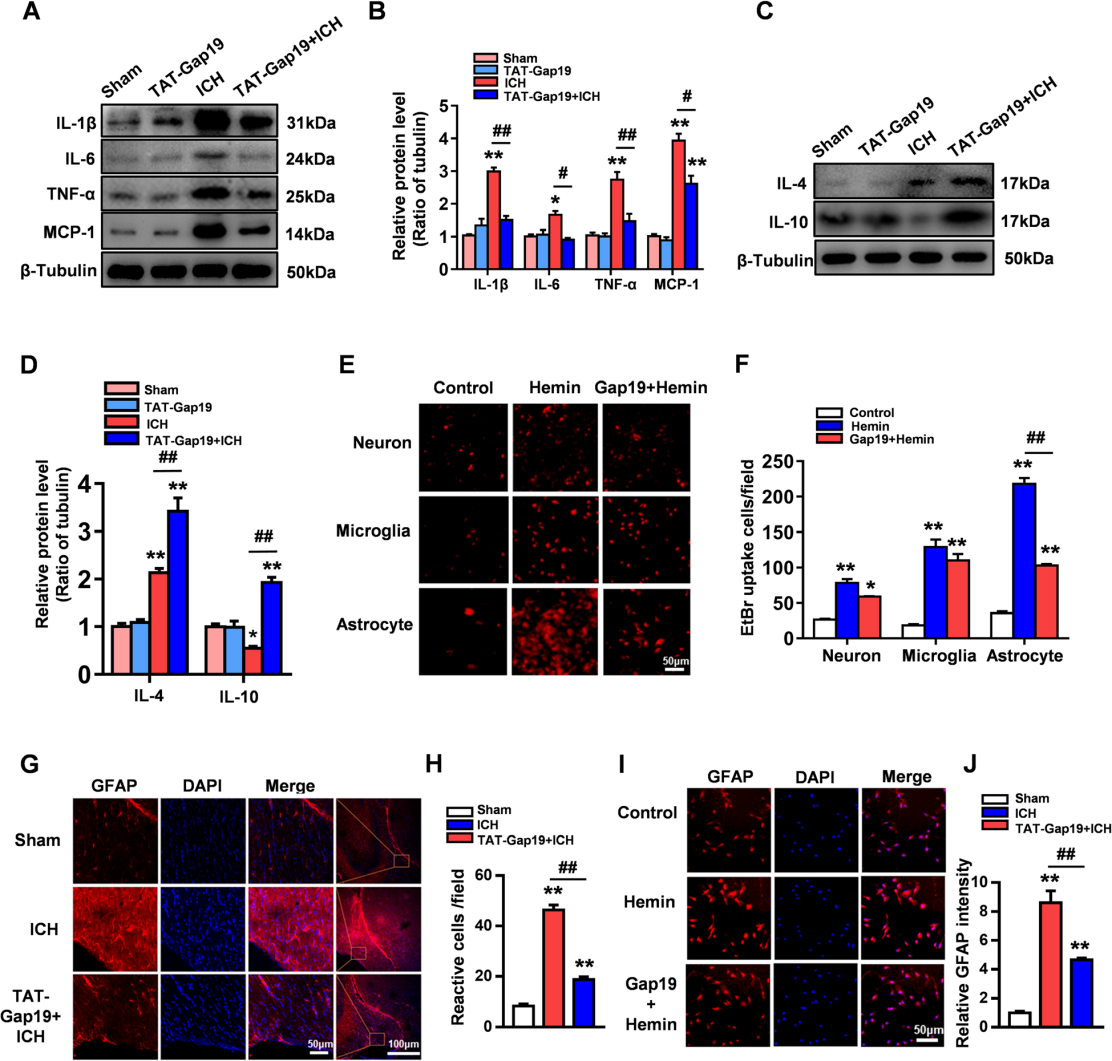
Gap19 reduces the expression of proinflammatory cytokines and the activation of astrocytes after ICH injury. **a**, **b** WB analysis showed the expression levels of TNF-α, IL-1β, IL-6, MCP1, and β-tubulin as the loading control. **c**, **d** The expression levels of IL-4 and IL-10. β-Tubulin was used as a loading control. **e**, **f** The EtBr uptake assay was used to evaluate the activity of Hcs on astrocytes, microglia, and neurons after hemin stimulation. Gap19 inhibits the hemin-induced excessive opening of Hcs on astrocytes. **g**, **h** Immunofluorescence for GFAP was used to evaluate astrocyte activation. TAT-Gap19 treatment significantly reduced the number of GFAP-positive cells in the area surrounding cerebral hemorrhage. **i**, **j** In vitro, Gap19 treatment reduced the GFAP fluorescence intensity in astrocytes after hemin stimulation. The bars in **a**–**d**, **g**, and **h** represent the SEM of the data from 3 cerebral hemorrhage tissue samples per group. *P < 0.05, **P < 0.01 compared to the sham group. #*P* < 0.05, ##*P* < 0.01 compared with the ICH group. The bars in **e**, **f**, **i**, **j** in the ICH group represent the SEM of data from 3 samples per group. *P < 0.05, **P < 0.01 compared to the control group. #*P* < 0.05, ##*P* < 0.01 compared with the hemin group

Inflammation is considered the core factor underlying secondary damage after ICH injury. Various proinflammatory cytokines and chemokines, including IL-6, IL-1β, TNF-α, and MCP-1, are produced and secreted into the brain parenchyma [26]. However, anti-inflammatory cytokines such as IL-4 and IL-10 are also released by immune cells following ICH-induced damage [27]. In a previous study, blocking Cx43Hcs with a Cx43 mimetic peptide was demonstrated to inhibit inflammation [28]. In this study, we examined the protein expression levels of proinflammatory cytokines 72 h after ICH injury, including IL-1β, IL-6, TNF-α, and MCP-1, and anti-inflammatory cytokines, including IL-4 and IL-10. After ICH injury, the levels of IL-1β, IL-6, TNF-α, and MCP-1 were markedly upregulated. Interestingly, compared to the ICH group, TAT-Gap19 not only decreased the expression levels of proinflammatory cytokines (IL-1β, IL-6, TNF-α, and MCP1) but also increased the levels of anti-inflammatory cytokines (IL-4 and IL-10) after ICH injury (*P* < 0.05, Fig. 3a–d).

### Gap19 reduces astrocyte activation and astrocytic proinflammatory cytokine production after ICH injury

Because Cx43 is most abundant in astrocytes and regulates the network between other cells in the CNS through gap junctions, we measured the pharmacological effects of Gap19 in blocking the activity of Cx43Hcs on different types of primary neurocytes, including astrocytes, microglia, and neurons, after 12 h of hemin stimulation. In culture, EtBr uptake by all three types of neurocytes was higher in the hemin-treated groups than in the control groups (*P* < 0.01, Fig. 3e, f). After hemin stimulation, Gap19 slightly blocked the opening of Hcs on neurons and microglia, but the difference was not significant (*P* > 0.05, Fig. 3e, f). However, compared to vehicle, Gap19 significantly inhibited the opening of astrocytic Hcs after hemin stimulation (*P* < 0.01, Fig. 3e, f). Based on these results, we explored whether abnormal Cx43Hc opening mainly occurs on the surface of astrocytes after ICH injury.

A previous study reported that Cx43 may be related to the activation of astrocytes and that Cx43 peptides can reduce the number of reactive astrocytes after ischemic stroke [23]. We labeled reactive astrocytes after ICH injury in vivo and in vitro by staining for GFAP. In mouse sections from the sham group, a small number of reactive astrocytes were observed in the peripheral area of the striatum; however, there was a significant increase in reactive astrocytes around the cerebral hematoma in the ICH group compared to the sham group 72 h after ICH injury (*P* < 0.01, Fig. 3g, h). Compared to control, treatment with TAT-Gap19 markedly reduced the number of reactive astrocytes compared with that in the ICH group (*P* < 0.01, Fig. 3g, h). We obtained consistent results in astrocytes subjected to hemin stimulation, which was used to simulate ICH injury in vitro. Compared to control, Gap19 treatment significantly reduced the GFAP fluorescence intensity, which was much higher in astrocytes stimulated with hemin for 12 h than in unstimulated astrocytes (*P* < 0.01, Fig. 3i, j). These results indicate that blocking astrocytic Cx43Hcs with Gap19 can reduce the number of reactive astrocytes after ICH injury.

**Fig. 4:**
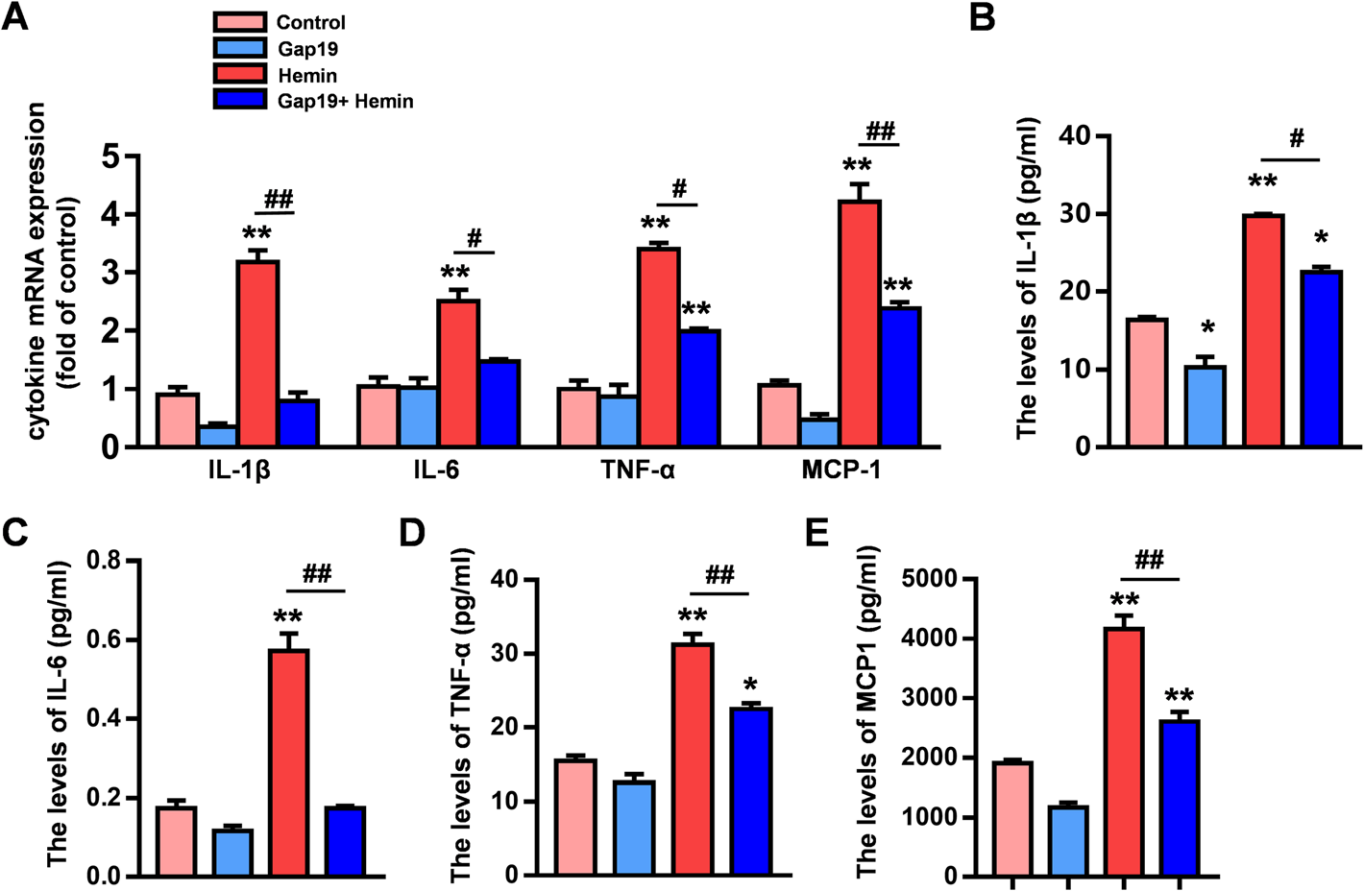
Gap19 reduces the production and release of proinflammatory cytokines by astrocytes after ICH injury. **a** qRT-PCR analysis of the transcriptional activities of IL-1β, IL-6, TNF-α, and MCP-1 in astrocytes. **b**–**e** ELISA was used to detect the release of IL-1β, IL-6, TNF-α, and MCP1 from astrocytes to the supernatant following different treatments. The bars represent the SEM of data from 3 samples per group. *P < 0.05, **P < 0.01 compared to the control group. #*P* < 0.05, ##*P* < 0.01 compared with the hemin group

Studies have shown that reactive astrocytes produce and release various proinflammatory cytokines and chemokines after brain injury [29]. In this study, the transcriptional activity of proinflammatory cytokines and chemokines, including IL-1β, IL-6, TNF-α, and MCP-1, in cultured astrocytes was measured by qRT-PCR 12 h after hemin stimulation. The transcriptional activities of IL-1β, IL-6, TNF-α, and MCP-1 were upregulated after hemin stimulation. In the hemin group, compared to control, Gap19 markedly alleviated the transcriptional activity of upstream proinflammatory cytokines and chemokines (*P* < 0.01, Fig. 4a). Furthermore, ELISA was employed to evaluate proinflammatory cytokine and chemokine release by cultured astrocytes after hemin stimulation. Much higher levels of IL-1β, IL-6, TNF-α, and MCP-1 were detected in the supernatants of the hemin group than in the supernatants of the control group. However, compared to control, Gap19 reduced the secretion of upstream proinflammatory cytokines and chemokines by astrocytes after hemin stimulation (*P* < 0.05, Fig. 4b–e). These findings further demonstrate that Gap19 exerts protective effects against ICH-induced inflammation.

### Gap19 promotes the degradation of astrocytic Cx43 through the ubiquitin-proteasome pathway after hemin stimulation

**Fig. 5:**
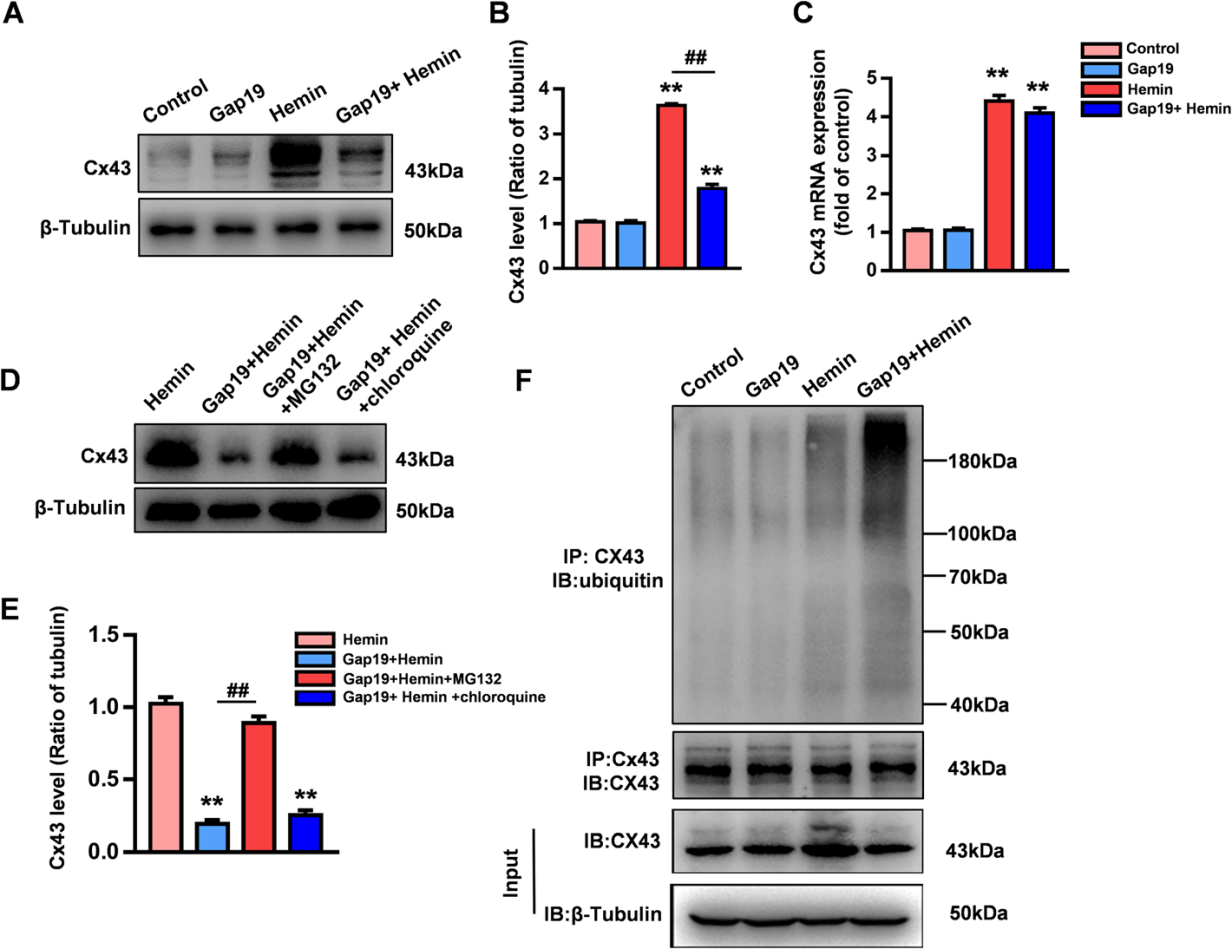
Gap19 promotes the degradation of astrocytic Cx43 through the ubiquitin-proteasome pathway after hemin stimulation. **a**, **b** WB analysis showed that Gap19 treatment reduced the Cx43 levels induced by hemin stimulation. **c** qRT-PCR analysis showed that after hemin stimulation, Gap19 treatment had no effect on hemin stimulation-induced Cx43 transcriptional activity. **d**, **e** Treatment of cultured astrocytes with MG132 and chloroquine for 12 h. WB analysis showed that MG132 increased the expression of Cx43 after Gap19 treatment (*P* < 0.05), but there was no significant difference in Cx43 levels between the chloroquine-treated group and the Gap19 + hemin group. **f** Coimmunoprecipitation with an anti-Cx43 antibody was used to analyze the ubiquitination of Cx43 in astrocytes after hemin stimulation. This figure shows that Gap19 treatment increased the level of Cx43 ubiquitination in astrocytes after hemin stimulation. The bars represent the SEM of the data from 3 samples per group. In **d** and **e**, *P < 0.05, **P < 0.01 compared to the hemin group. #*P* < 0.05, ##*P* < 0.01 compared with the Gap19 + hemin group. In the rest of the figures, *P < 0.05, **P < 0.01 compared to the control group. #*P* < 0.05, ##*P* < 0.01 compared to the hemin group

To explore the effects of Gap19 treatment on astrocytic Cx43 after ICH injury, we used WB and qRT-PCR to detect the expression level of astrocytic Cx43 in cultured cells after hemin stimulation. Total protein and RNA were extracted from astrocytes cultured under different conditions. Both the protein level and transcriptional activity of Cx43 were markedly increased in the hemin group compared with the control group (*P* < 0.01, Fig. 5a–c). At the same time, Western blot analysis further confirmed that compared to no treatment, Gap19 treatment reduced the overexpression of Cx43 induced by hemin stimulation (*P* < 0.01, Fig. 5a, b). However, Gap19 decreased the protein level but not the transcriptional activity of astrocytic Cx43 compared with that in the hemin group (*P* > 0.05, Fig. 5c). Thus, Gap19 may affect the stability of Cx43 in astrocytes after ICH injury, which is in agreement with the effects of Gap19 on cerebral ischemia injury observed in our prior study [25].

There is sufficient evidence showing that ubiquitination plays a key role in Cx43 degradation, which contributes to functional changes in gap junction channels. Studies have found that Cx43 can be rapidly degraded either by lysosomes or through the ubiquitin-proteasome pathway [30]. To further evaluate the degradation mechanism of astrocytic Cx43 after hemin stimulation, the proteasome inhibitor MG132 and lysosomal inhibitor chloroquine were used. WB analysis showed that MG132 treatment almost reversed the effect of Gap19 on astrocytic Cx43 degradation after hemin treatment (*P* < 0.01), while chloroquine did not have any significant anti-degradation effects (Fig. 5d, e). We further detected the ubiquitination of astrocytic Cx43 after hemin stimulation by conducting immunoprecipitation with an anti-Cx43 antibody and anti-ubiquitin antibody to clarify the process by which Cx43 is ubiquitinated. Cx43 ubiquitination was enhanced by Gap19 treatment in hemin-stimulated astrocytes (Fig. 5f). These results indicate that Gap19 promotes Cx43 degradation through the ubiquitin-proteasome system in an in vitro model of ICH injury.

### Gap19 increases the activity of YAP in astrocytes after ICH injury in vitro

**Fig. 6:**
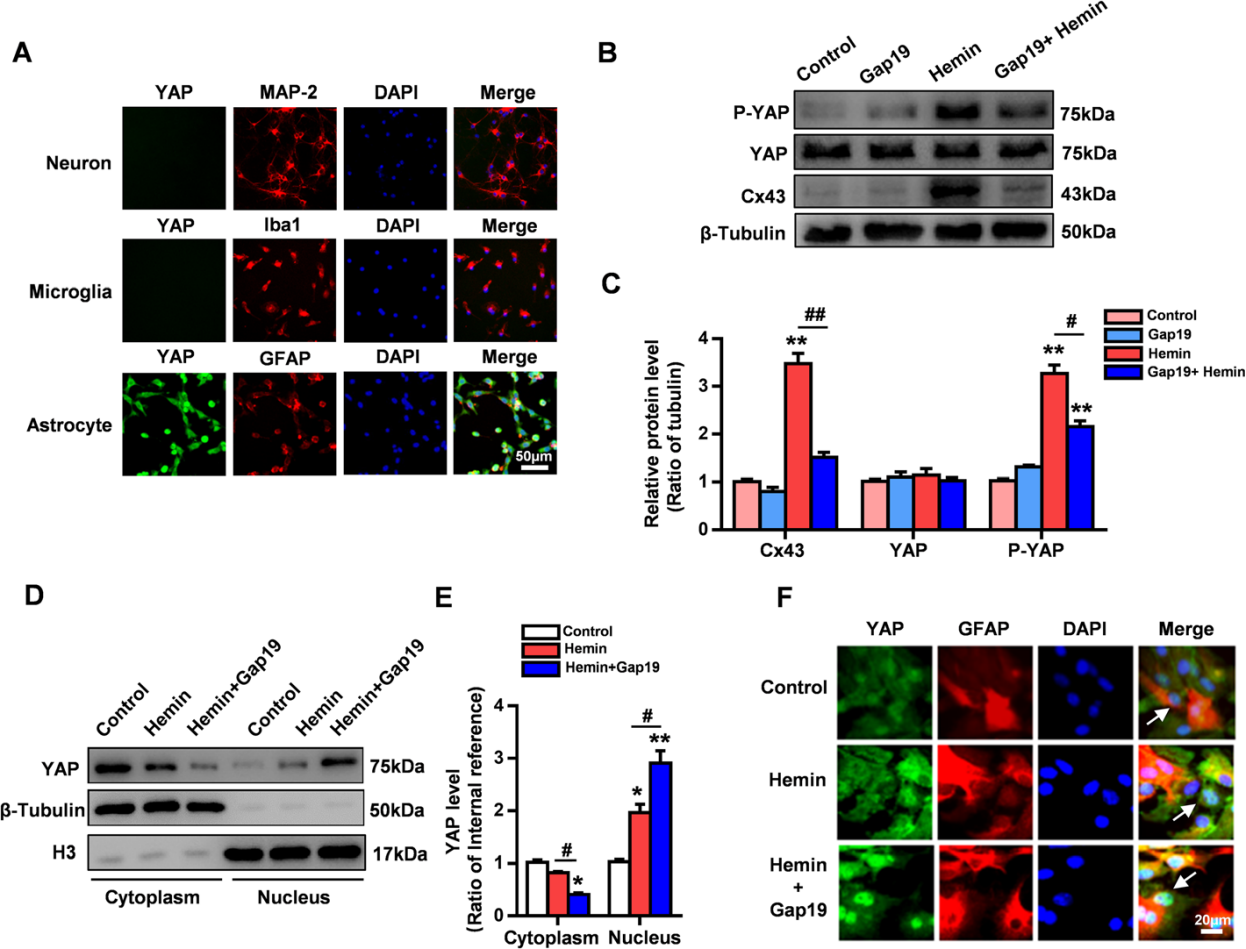
Gap19 increases the activity of YAP in astrocytes exposed to ICH injury. **a** Immunofluorescence staining of astrocytes, microglia and neurons revealed that YAP was mainly expressed in astrocytes. **b**, **c** Gap19 was administered to astrocytes at the beginning of hemin stimulation. Proteins were extracted 12 h after hemin stimulation, and the levels of Cx43, YAP, and p-YAP were determined by WB analysis. Representative photos showing the levels of Cx43, YAP, p-YAP, and β-tubulin. **d**, **e** Quantitative WB analysis of YAP expression in the astrocyte cell cytoplasm and nucleus. The bars represent the SEM of the data from 3 samples per group. **f** Immunofluorescence showing that YAP nuclear translocation increased after treatment with Gap19 after hemin stimulation in astrocytes. *P < 0.05, **P < 0.01 compared to the control group. #*P* < 0.05, ##*P* < 0.01 compared to the hemin group

A previous study showed that Cx43 may regulate YAP nuclear translocation in cultured astrocytes after hemoglobin stimulation [31]. Furthermore, YAP deletion has been demonstrated to lead to excessive activation of astrocytes during the development of the nervous system [32]. Additionally, prior research has demonstrated that YAP can be sequestered at the cellular junction and may regulate cell-cell interactions [18]. We speculate that the pharmacological action of Gap19 in inhibiting the activation of astrocytes, which results in anti-inflammatory effects, may be associated with the interaction between astrocytic Cx43 and YAP after ICH injury. Our immunofluorescence staining results showed that YAP was mainly expressed in astrocytes but that there was almost no YAP expression in neurons and microglia (Fig. 6a); this is consistent with the findings of Zhihui Huang et al. [32]. The activity of YAP depends on its phosphorylation state. The expression levels of YAP and phosphorylated YAP in astrocytes after hemin stimulation were detected by WB. In addition to causing Cx43 overexpression, hemin stimulation significantly increased phosphorylated YAP expression levels in astrocytes compared to those in the control group (*P* < 0.01, Fig. 6b, c). Furthermore, we evaluated the expression of YAP in the nucleus and cytoplasm. The data confirmed our hypothesis that Gap19 significantly increases nuclear YAP expression and decreases cytoplasmic YAP expression (*P* < 0.05, Fig. 6d, e). Immunofluorescence staining showed a marked increase in YAP nuclear translocation after Gap19 treatment (Fig. 6f). These results indicate that the Cx43 mimetic peptide Gap19, which selectively blocks Cx43Hcs, may inhibit the proinflammatory function of reactive astrocytes by regulating the activity of YAP in hemin-stimulated astrocytes.

### Gap19 physically regulates the association between astrocytic Cx43 and YAP in the cytoplasm after hemin stimulation

**Fig. 7:**
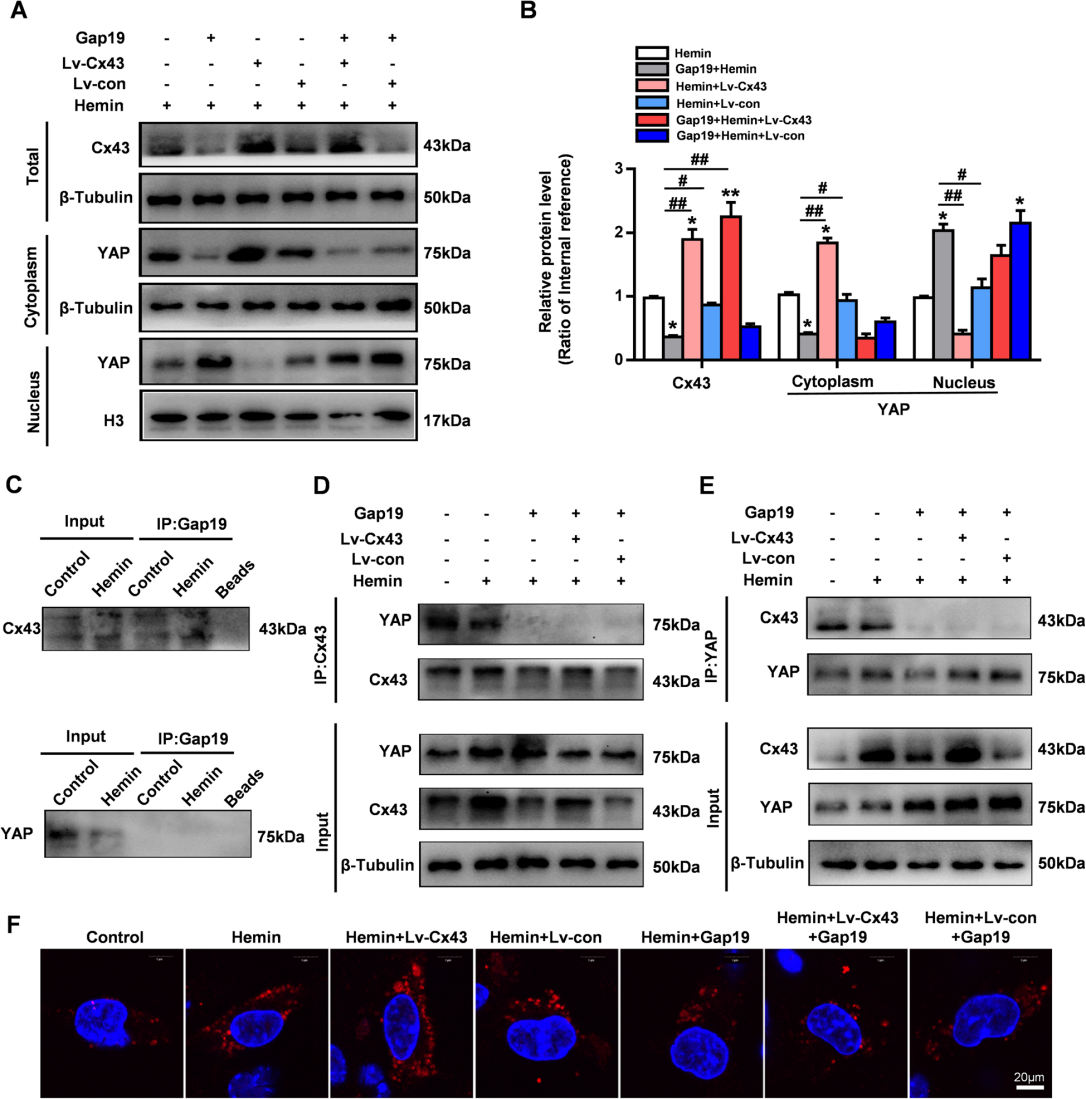
Gap19 regulates the physical association between astrocytic Cx43 and YAP in the cytoplasm after hemin stimulation. **a**, **b** Cx43 was overexpressed by lentiviral transfection, and the levels of cytoplasmic/nuclear YAP were measured by WB in hemin-stimulated astrocytes. Quantitative analysis showed that Lv-Cx43 transfection decreased nuclear YAP translocation after hemin stimulation. However, Cx43 overexpression had no effects on YAP nuclear translocation after Gap19 treatment. **c** Coimmunoprecipitation experiments showing that Cx43 interacted with Gap19 but not YAP in hemin-stimulated astrocytes. **d**, **e** Coimmunoprecipitation experiments showing that YAP interacted with Cx43 and that Gap19 interfered with the binding of astrocytic Cx43 to YAP in the cytoplasm after hemin stimulation. **f** Representative images of the Duolink in situ PLA showing that there was a direct interaction between YAP and Cx43, that the effect was enhanced after Cx43 overexpression by Lv-Cx43, and that the interaction was weakened after Gap19 treatment. The bars represent the SEM of the data from 3 samples per group. *P < 0.05, **P < 0.01 compared to the hemin group. #*P* < 0.05, ##*P* < 0.01 compared with the Gap19 + hemin group

To further clarify the Cx43-YAP interaction in hemin-stimulated astrocytes, we overexpressed Cx43 with lentivirus to observe the subsequent changes in YAP nuclear translocation under different experimental conditions. Compared with LV-con, Lv-Cx43 effectively upregulated the expression of Cx43 and reversed the inhibition of Cx43 expression by Gap19 treatment (Fig. 7a, b). Consistent with a prior study, overexpression of Cx43 significantly increased the cytoplasmic expression of YAP while decreasing nuclear YAP levels (*P* < 0.01, Fig. 7a, b). Intriguingly, after Gap19 treatment, Lv-Cx43 did not effectively inhibit YAP nuclear translocation in hemin-stimulated astrocytes (*P* > 0.05, Fig. 7a, b). These results suggest that Gap19 may intervene in the association between astrocytic Cx43 and YAP in the cytoplasm under pathological conditions. Gap19-biotin was employed in our study to pull down bound proteins with anti-biotin magnetic beads. The results confirmed that astrocytic Cx43 but not YAP interacted with Gap19 (Fig. 7c). To evaluate whether Gap19 disturbs the physical association between astrocytic Cx43 and YAP in the cytoplasm after hemin stimulation, a coimmunoprecipitation assay and PLA were performed. After extracting astrocytic cytoplasmic proteins, we used anti-Cx43 and anti-YAP antibodies to pull down bound proteins. Coimmunoprecipitation assays indicated that Cx43 directly bound to YAP in astrocytes cytoplasm but that Gap19 almost completely disrupted their interaction (Fig. 7d, e). Furthermore, PLA confirmed that the interaction between Cx43 and YAP in the astrocyte cytoplasm was enhanced by hemin stimulation and Cx43 overexpression. Gap19 significantly inhibited the above association, as evidenced by the significantly decreased intensity of red fluorescence in Gap19-treated astrocytes compared to control astrocytes (Fig. 7f). These results indicate that Gap19 plays a different role in disturbing the physical association between astrocytic Cx43 and YAP in the cytoplasm after hemin stimulation.

### Gap19 regulates the Cx43-YAP-SOCS axis in reactive astrocytes after ICH injury

Recent evidence has illustrated that the YAP-SOCS1/SOCS3 axis inactivates proinflammatory pathways in reactive astrocytes and regulates reactive astrogliosis under pathological conditions [32]. In this study, we detected the expression levels of SOCS1 and SOCS3 in cultured astrocytes. The results showed that the expression of SOCS1 and SOCS3 was increased in the hemin groups compared with the control group or Gap19 group (*P* < 0.05, Supplementary Fig. 1 A, B). However, Gap19 treatment significantly increased the expression levels of SOCS1 and SOCS3 compared with those in the hemin group (*P* < 0.01, Supplementary Fig. 1 A, B). Furthermore, the core markers of the TLR4-NFκB and JAK2-STAT3 pathways, which are known to be inactivated by SOCS1 and SOCS3, were measured by WB. Intriguingly, Gap19 downregulated the overexpression of TLR4, p-IKKβ, and p-p65 following hemin stimulation (*P* < 0.01, Supplementary Fig. 1 C, D). Additionally, compared to no treatment, Gap19 downregulated the overexpression of p-JAK2 and p-STAT3 in hemin-stimulated reactive astrocytes (*P* < 0.01, Supplementary Fig. 1 E, F).

**Fig. 8:**
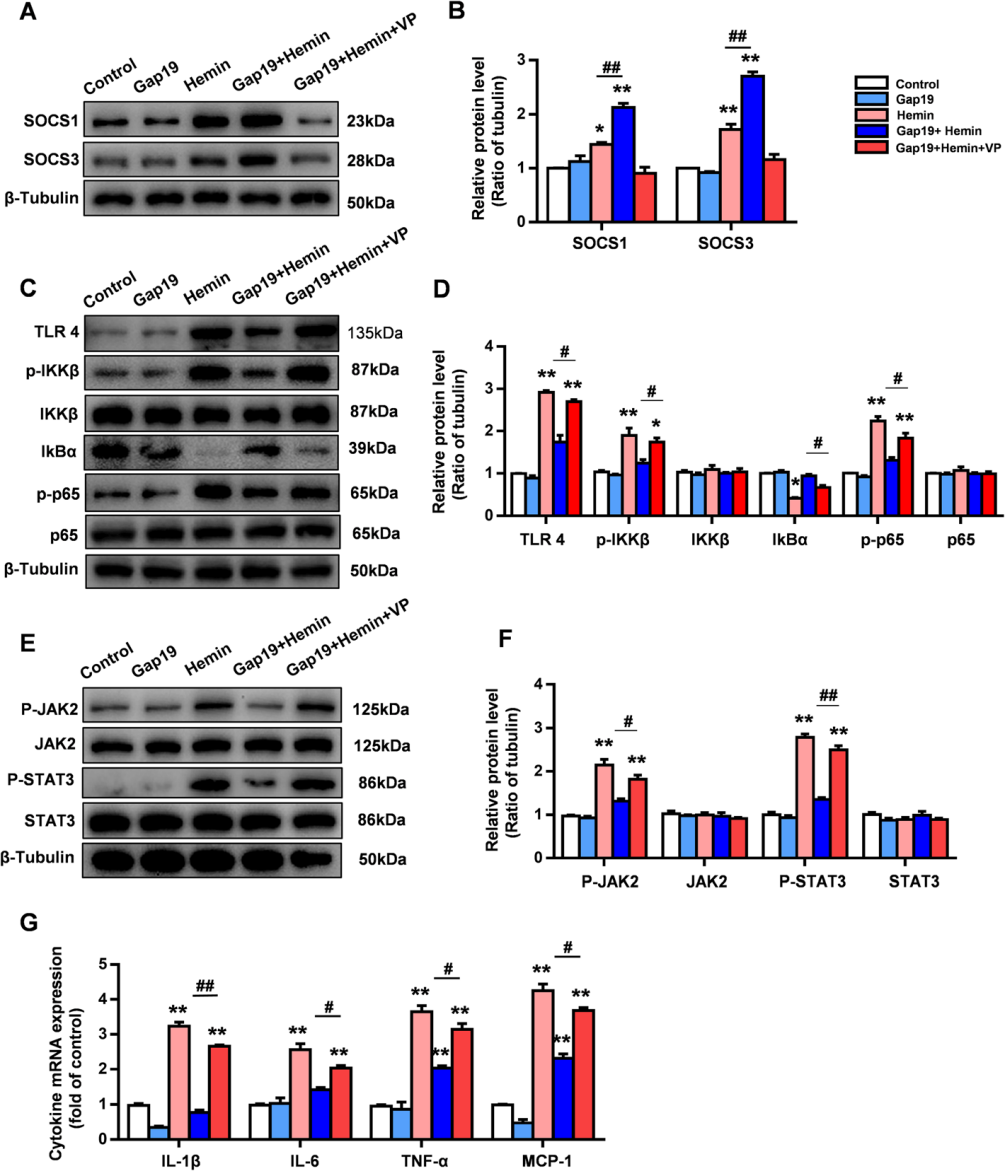
In vitro, the YAP inhibitor VP reverses the regulatory effect of Gap19 on the Cx43-YAP-SOCS axis. **a**, **b** At the beginning of hemin stimulation, astrocytes were treated with the YAP inhibitor VP. WB analysis showed that VP suppressed the increase in SOCS1/SOCS3 levels induced by Gap19 treatment. **c**, **d** Representative photos showing the levels of TLR4, p-IKKβ, IKKβ, IKBα, p65, p-p65, and β-tubulin after treatment with the YAP inhibitor VP. **e**, **f** Representative photographs showing the levels of JAK2, p-JAK2, STAT3, p-STAT3, and β-tubulin after treatment with the YAP inhibitor VP. **g** qRT-PCR analysis of the transcriptional activities of IL-1β, IL-6, TNF-α, and MCP1 in astrocytes after VP treatment. The bars represent the SEM of the data from 3 samples per group. *P < 0.05, **P < 0.01 compared to the hemin group. #*P* < 0.05, ##*P* < 0.01 compared with the Gap19 + hemin group

To further investigate whether the YAP-SOCS1/SOCS3 axis mediates the protective effects of Gap19, the widely accepted YAP inhibitor VP was employed. Previous studies have explored whether VP inhibits reactive astrogliosis in a dose-dependent manner after ICH injury. VP (5 μM/mL) was administered in vitro at the beginning of hemin stimulation. Consistent with our hypothesis, VP reversed the increase in astrocytic SOCS1/SOCS3 levels induced by Gap19 after hemin stimulation (*P* < 0.05, Fig. 8a, b). Mechanistically, the ability of Gap19 to activate the TLR4-NFκB and JAK2-STAT3 pathways was blocked by VP interference (*P* < 0.05, Fig. 8c–f). Furthermore, the transcriptional activity of inflammatory cytokines and chemokines, including IL-1β, IL-6, TNF-α, and MCP-1, were significantly upregulated in the Gap19 + hemin + VP group compared with the Gap19 + hemin group (Fig. 8g). These results suggest that Gap19 inhibits inflammatory responses and astrocyte activation via the Cx43-YAP-SOCS1/SOCS3 axis to inhibit the TLR4-NF-κB and JAK2-STAT3 pathways in reactive astrocytes after hemin stimulation.

### The YAP inhibitor VP reverses the protective effect of Gap19 after ICH injury

**Fig. 9:**
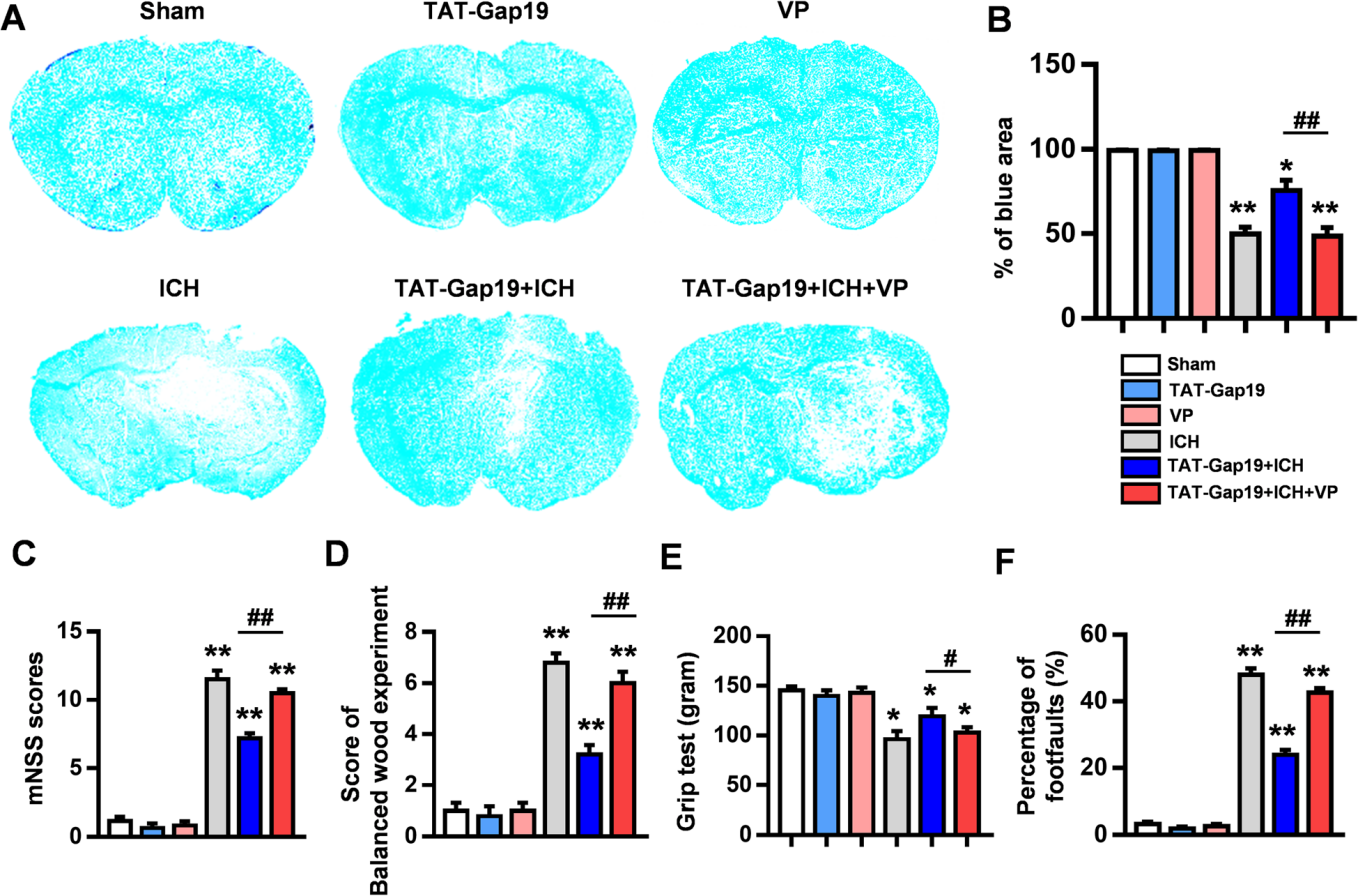
In vivo, the YAP inhibitor VP reverses the protective effect of Gap19 against the pathological process of ICH injury. **a**, **b** Representative photos of LFB staining showing that the volume of cerebral hematoma in the TAT-Gap19 + ICH + VP group was increased compared with that in the TAT-Gap19 + ICH group and that VP reversed the protective effect of Gap19 against white matter damage after ICH. **c**–**f** The mNSSs, balance beam scores, foot fault rates, and forelimb grip strength of mice treated with the YAP inhibitor VP were evaluated and statistically analyzed. The represent the SEM of the data from 6 samples per group. *P < 0.05, **P < 0.01 compared to the sham group. #*P* < 0.05, ##*P* < 0.01 compared with the TAT-Gap19 + ICH group

In the in vivo ICH model, we treated mice with VP (0.1 mg/kg body weight, injected into the lateral ventricle once a day for 3 consecutive days). Hematoma volume and behavior were evaluated after ICH damage. There was no brain damage or neurological deficits in the sham, Gap19, and VP groups (*P* > 0.05, Fig. 9a–f). As expected, VP treatment almost abolished the neuroprotective effect of Gap19 against ICH injury. LFB staining showed that compared with Gap19 treatment alone, inhibition of YAP signaling by VP increased the hematoma volume and reduced the blue-stained area in the mouse brain sections (*P* < 0.05, Fig. 9a, b). The neurobehavioral assessments showed that mNSSs, balance beam scores, and foot fault rates were increased and that grip strength of the forelimbs was decreased in the TAT-Gap19 + ICH + VP group compared with the TAT-Gap19 + ICH group (Fig. 9c–f). These data indicate that the anti-inflammatory and neuroprotective effects of the Cx43 mimetic peptide Gap19 after ICH injury in vitro and in vivo are mediated by YAP signaling.

## Discussion

Gap junctions are specialized cell-to-cell contacts that enhance crosstalk between cells and the pericellular space [33]. In the CNS, GJCs, composed of two Hcs on each cell, allow cytoplasmic exchange of multiple substances between adjacent cells [34]. Importantly, the opening and closing of GJCs or Hcs depend on their response to stimuli such as other cellular membrane channels [35]. Connexin proteins are the necessary components of GJCs and Hcs, and Cx43 is one of the most abundant members of the connexin protein family [36]. In recent years, studies have indicated that Cx43-containing GJCs and Hcs are the main cellular adhesion channels in astrocytes and maintain homeostasis of the neural microenvironment [22]. However, under pathologic conditions, the abnormal opening of Cx43Hcs on the surface of reactive astrocytes can release small molecules into the intercellular space [37]. These small molecules influence the steady state of the cell microenvironment, inducing pro-inflammatory and cell death programs by regulating the crosstalk between reactive astrocytes and other cells [38]. Inhibition of Cx43Hcs with selective blockers has been shown to provide protective effects under different pathological stresses [39]. Our previous research confirmed that Cx43Hc blockers significantly alleviate neurological deficits and infarct volume in a rodent model of ischemic stroke [10, 40]. To our knowledge, there have been no reports on the characteristics of Cx43Hcs after ICH injury. In our current study, sustained overexpression of Cx43 coupled with abnormal opening of Cx43Hcs was observed in a mouse model of ICH injury (Fig. 1). Delayed treatment with the selective Cx43Hc blocker TAT-Gap19 for 3 days after ICH injury significantly blocked the excessive opening of Cx43Hcs and reduced neuronal apoptosis and white matter damage. Gap19 significantly alleviated the hematoma volume and neurological deficits after ICH injury (Fig. 2). This is the first study to demonstrate that inhibiting abnormal Cx43Hc opening is a feasible strategy for treating ICH injury.

Although multiple pathological mechanisms are involved in ICH, increasing evidence has demonstrated that the inflammatory response is the main contributor toward ICH progression [41]. Inflammatory mediators, such as cytokines and chemokines, are released and aggravate brain damage through the inflammatory cascade, resulting in the further recruitment and activation of immune cells during ICH [42]. Therefore, inhibiting the neuroinflammatory cascade has become an effective therapeutic strategy for treatment of ICH injury. Inflammatory factors such as ATP, NADH, and PGE2 are released into the extracellular space through Cx43Hcs, leading to the activation of intracellular inflammatory pathways [43]. Cx43 mimetic peptides or Cx43 antisense oligodeoxynucleotides in addition to partial Cx43 deletions inhibit spinal cord injury by reducing proinflammatory cytokine levels [44]. Consistent with these results, we found that proinflammatory mediators, including IL-1β, IL-6, TNF-α, and MCP-1, were downregulated in TAT-Gap19-treated ICH mice compared to untreated mice, while anti-inflammatory cytokines, including IL-4 and IL-10, were upregulated (Fig. 3). Taken together, these results suggest that ICH injury induces abnormal excessive Cx43Hc opening, which causes an increase in neuroinflammatory responses, and that this elevation is reversed by Gap19 treatment.

Recently, reactive astrocytes were identified to be present in the CNS under pathological environment [45, 46]. The immune response is aggravated by reactive astrogliosis following various types of CNS injury, including ICH, due to their ability to secrete chemokines and cytokines that diffuse from the injury site to the surrounding regions [47, 48]. Microscopically, reactive astrocytes influence the activation of other immune cells, including microglia and macrophages, to increase the inflammatory cascade in the neural microenvironment [49]. Additionally, reactive astrocytes play a vital role in crosstalk with microglia by releasing cytokines, chemokines, and small molecules that influence the inflammatory response after ICH injury [50]. Therefore, inhibiting the proinflammatory function of reactive astrocytes and the inflammatory cascade that results from acute astrocyte activation can alleviate inflammatory responses after ICH injury. Cx43, the most abundant gap junction protein in astrocytes, regulates astrogliosis function in the CNS [12]. Cx43 overexpression coupled with excessive Cx43Hc opening contributes to astrocyte reactive migration, leading to increased neuroinflammation [51]. Vignal N et al. reported that astrocytic Cx43 deficiency protects against lipopolysaccharide (LPS)-induced CNS [52]. Kim Y et al. demonstrated that blocking astrocytic Cx43Hcs with tonabersat can significantly inhibit local inflammation in the CNS [53]. Additionally, CX43 upregulation, which activates astrocytic Hcs, has been observed under inflammatory conditions in the hSOD^G93A^ ALS mouse model. Additionally, pharmacological blockade of Cx43 by Hc blockers reduces the inflammatory response and exerts protective effects in cocultured motor neurons and hSOD^G93A^ astrocytes [54]. In this study, abnormal Cx43 overexpression and excessive Cx43Hc opening were mainly detected in astrocytes rather than neurons or microglia after hemin stimulation *in vitro* (Figs. 3 and 5). In addition to reversing Cx43 overexpression and excessive Cx43Hc opening in astrocytes, Gap19 significantly decreased astrocyte activation after ICH injury in vitro and in vivo, as determined by staining for GFAP, a hallmark of reactive astrogliosis (Fig. 3). Furthermore, Gap19 treatment reduced proinflammatory cytokine production and release by reactive astrocytes (Fig. 4). Consistent with our previous study in a mouse model of cerebral ischemic stroke, our present data demonstrated that astrocyte activation and proinflammatory effects after ICH injury were regulated by Cx43 or Cx43Hcs. However, the mechanism by which astroglial function is altered by the Cx43 mimetic peptide needs to be further evaluated.

Ubiquitination is an important pathway underlying membrane protein degradation in both physiological and pathological states [55]. Conclusively, the degradation of Cx43 by the ubiquitin-proteasome system has been demonstrated to determine the fate of gap junctions [56]. The proteasomal degradation of Cx43 was first explored by evaluating the effects of the proteasome inhibitor MG132, which enhances the stabilization of Cx43 at the plasma membrane [57]. However, Cuervo et al. showed that macroautophagy contributes to the ubiquitination of Cx43 during serum starvation [58]. This discrepancy may have resulted from the induction of different types of posttranslational modification of Cx43 by different stimuli. In our previous study, Cx43 mimetic peptides, including Gap26 and Gap27, were shown to increase the degradation of astrocytic Cx43 through the ubiquitin-proteasome pathway in culture after oxygen-glucose deprivation [59, 60]. We also showed that Gap19 has pharmacological effects in reducing the expression levels of astroglial Cx43 after cerebral ischemia injury. In this study, the transcriptional activity and expression of Cx43 in astrocytes were significantly increased in culture after hemin stimulation. However, after hemin stimulation, Gap19 treatment downregulated the expression of astrocytic Cx43 but did not influence the transcriptional activity of Cx43, revealing that Gap19 reduced the stability of Cx43. As expected, Gap19 enhanced the ubiquitination of astrocytic Cx43 after hemin stimulation. Interestingly, the downregulation of astrocytic Cx43 by Gap19 was reversed by MG132 but not chloroquine (a lysosomal inhibitor), which suggests that the ubiquitin-proteasome pathway may contribute to the abovementioned pharmacological phenomenon. These results strongly suggest that Cx43 ubiquitination was increased by Gap19 treatment (Fig. 5). However, the detailed mechanism of how Gap19 regulates the ubiquitination of astrocytic Cx43 after ICH injury still requires further study. We speculate that Gap19, as a mimetic peptide of Cx43, may influence the sites of astrocytic Cx43 posttranslational modification under pathologic conditions.

The proinflammatory mechanisms of astrocytic Cx43 in CNS diseases are still unclear. However, increasing evidence has demonstrated that blockers of Cx43Hcs can regulate many intracellular signaling pathways. Yin et al. showed that carbenoxolone and Gap26, blockers of Cx43Hcs, significantly inhibit neuroinflammation in an astrocytic ischemia model by regulating PKC, PKB, and Src signaling in both the membrane and cytoplasm [28]. Li et al. found that the Cx43 mimetic peptides Gap26 and Gap27 promote cytoplasmic degradation of astroglial Cx43 via the PI3K/Akt pathway in neonatal rats after ischemic brain injury [40]. Our previous study also found that Gap19 exerts its anti-inflammatory effects by inhibiting the activation of the JAK2/STAT3 and TLR4 signaling pathways in astrocytes after cerebral ischemic stroke [10, 25]. Consistent with these results, we believe that modulating intracellular signaling pathways may change the function of astrocytic Cx43 on the cellular plasma membrane.

The Hippo signaling pathway plays a key role in tissue homeostasis, tissue regeneration, and organ size regulation. Early studies showed that abnormal Hippo signaling contributes to tumorigenesis [61]. Recently, mounting evidence has shown the important role of Hippo/YAP signaling in CNS homeostasis. Dysfunction of Hippo/YAP signaling contributes to negative proapoptotic and proinflammatory effects in CNS diseases [15]. During cerebral ischemic injury, YAP deletion aggravates BBB and astrocyte damage. MST1, another important regulator of YAP in the Hippo pathways, is overactivated in a model of ICH, and knocking down MST1 mitigates inflammatory reactions and neuronal cell death [62]. Recently, the functions of YAP signaling in astrocytes were evaluated. Abnormal reactive astrogliosis and microglial activation are observed in YAP knockout mice during cerebral development. Hence, astroglial YAP deletion leads to excessive activation of inflammatory pathways, which potentiates the proinflammatory effects of reactive astrogliosis [32, 63]. YAP has also been shown to play an important role in regulating cellular junctions. Inhibition of its transcriptional activity at the plasma membrane can disrupt cellular contacts [64]. Yang et al. found that astrocytic Cx43 reduces the nuclear translocation of YAP in a hemoglobin mouse model [31]. These studies suggest that the proinflammatory functions of astroglial Cx43 and Cx43Hcs may be associated with YAP signaling in ICH injury. Our results first showed that after ICH, YAP expression was more abundant in astrocytes than in neurons and microglia. Cx43 overexpression was accompanied by an increased level of phosphorylated YAP in cultured reactive astrocytes after hemin stimulation. In contrast, Gap19 decreased astroglial Cx43 levels, decreased the level of phosphorylated YAP, and significantly increased YAP nuclear translocation (Fig. 6). We speculated that the protective effects of Gap19 may be mainly associated with its ability to regulate the degradation of cytoplasmic Cx43, which triggers YAP nuclear translocation in cultured astrocytes. However, surprisingly, Cx43 overexpression did not reverse YAP nuclear translocation after Gap19 treatment. Coimmunoprecipitation and PLA revealed that Gap19 interrupts the physical association between Cx43 and YAP in the astrocytic cytoplasm. To our knowledge, this is the first investigation to explore the interaction between Cx43 and YAP following Gap19 treatment (Fig. 7). These data indicate that the functional changes in astrocytic Cx43 and Cx43Hcs may be associated with the Cx43-YAP interaction, which contributes to astrocyte activation and regulates the inflammatory response after ICH injury.

Recent evidence has shown that the transcriptional activity of SOCS family members, including SOCS1 and SOCS3, in astrocytes is regulated by YAP signaling [32]. Multiple studies have confirmed that SOCS1 and SOCS3 overexpression inhibits the inflammatory TLR4-NFκB and JAK-STAT pathways in CNS diseases [65]. In this study, following hemin stimulation, the expression of SOCS1 and SOCS3 in astrocytes was decreased, while the inflammatory TLR4-NFκB and JAK2-STAT3 pathways were activated. Gap19 reversed the downregulation of the two proteins in hemin-stimulated astrocytes. The inflammatory TLR4-NFκB and JAK2-STAT3 pathways were also inhibited by Gap19 treatment (Fig. 8). Finally, VP, a nuclear YAP activity inhibitor, not only reversed the anti-inflammatory effect in vitro but also almost completely blocked the protective effects of Gap19 in vivo after ICH injury (Figs. 8 and 9). Therefore, our results reveal that the protective effects of Gap19 in blocking Cx43Hcs may be associated with functional changes in the Cx43-YAP-SOCS axis in reactive astrocytes after ICH injury.

## Conclusion

In summary, our findings are the first to demonstrate that abnormal Cx43 overexpression and excessive Cx43Hc opening aggravate brain tissue damage and neurological deficits after ICH injury. The Cx43 mimetic peptide Gap19 has neuroprotective effects in an ICH animal model. Gap19 suppresses abnormal astroglial Cx43 expression and Cx43Hc opening, which inhibits astrocyte activation and pro-inflammatory YAP signaling after ICH injury. This study provides new insight into potential treatment strategies for ICH injury involving astroglial Cx43 and Cx43Hcs.

## Supplementary information

**Additional file 1:.** Supplement Fig 1. Gap19 regulates the Cx43-YAP-SOCS axis in reactive astrocytes after ICH injury. (A, B) Representative pictures showing the levels of SOCS1, SOCS3, and β-tubulin. (C, D) Representative pictures showing TLR4, p-IKKβ, IKKβ, IKBα, p65, p-p65, and β-tubulin levels. (E, F) Representative pictures show the levels of JAK2, p-JAK2, STAT3, p-STAT3, and β-tubulin. The bars represent the SEM of the data from 3 samples per group. *, P<0.05, ** P<0.01 compared to the hemin group. #, P<0.05, ##, P<0.01 compared with the Gap19+hemin group.

## Data Availability

All data generated or analyzed during this study are included in this published article and its supplementary information files.

## Abbreviations

CNS: Central nervous system
Cx43: Connexin43
Cx43Hcs: Cx43 hemichannels
Co-IP: Co-immunoprecipitation
EtBr: Ethidium bromide
FFT: Foot fault test
FJC: Fluoro-Jade C
GJCs: Gap junction channels
ICH: Intracerebral hemorrhage
LFB: Luxol fast blue
mNSS: Modified neurological severity score
PLA: Proximity ligation assay
VP: Verteporfin
