# Supplementary figures and images for: Targeting connexin 43 provides anti-inflammatory effects after intracerebral hemorrhage injury by regulating YAP signaling
Source: J Neuroinflammation. 2020 Oct 28;17:322. doi: 10.1186/s12974-020-01978-z (PMC7594305; doi:10.1186/s12974-020-01978-z)

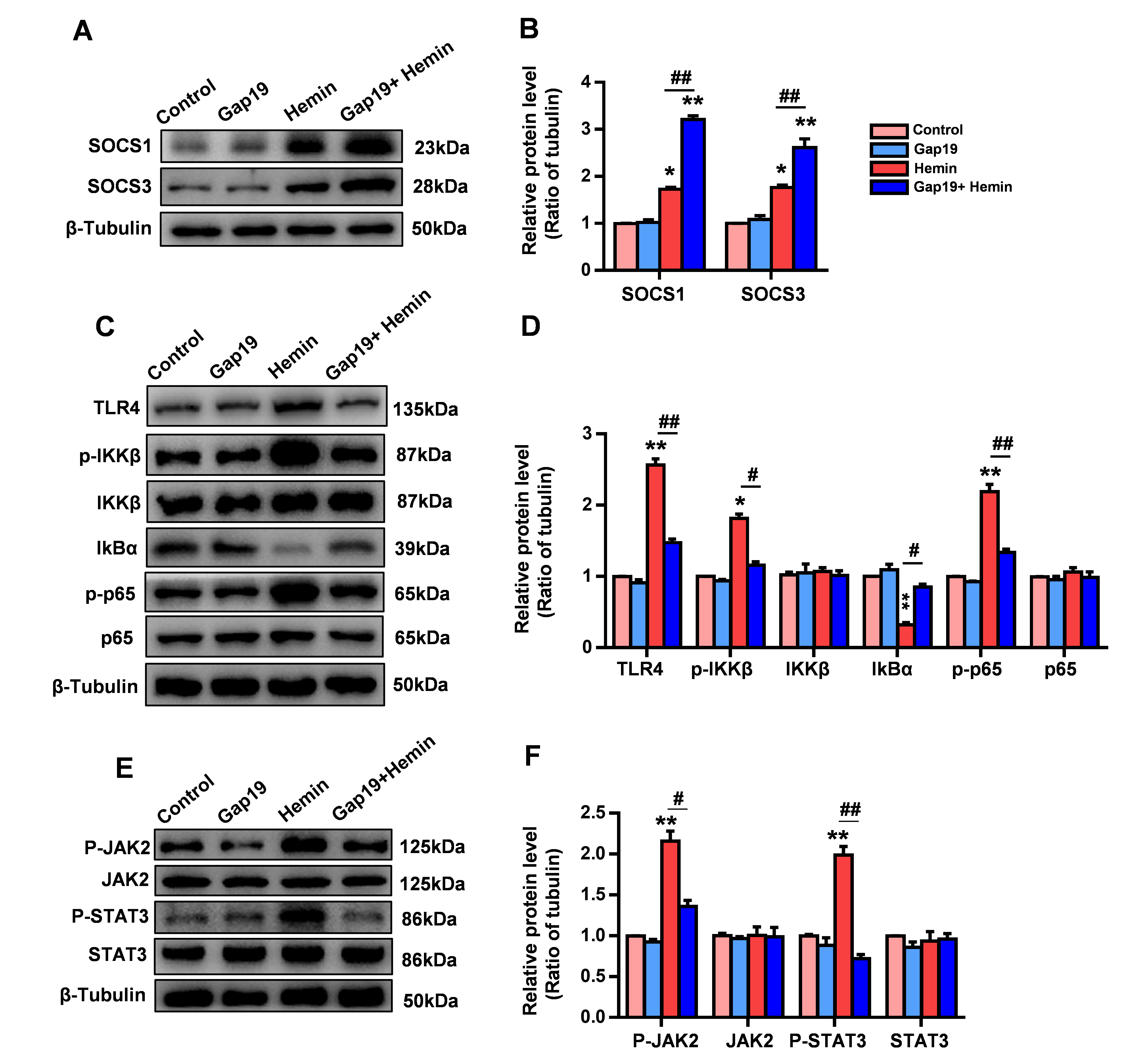

Supplement: Supplementary file 1 — Additional file 1:. Supplement Fig 1. Gap19 regulates the Cx43-YAP-SOCS axis in reactive astrocytes after ICH injury. (A, B) Representative pictures showing the levels of SOCS1, SOCS3, and β-tubulin. (C, D) Representative pictures showing TLR4, p-IKKβ, IKKβ, IKBα, p65, p-p65, and β-tubulin levels. (E, F) Representative pictures show the levels of JAK2, p-JAK2, STAT3, p-STAT3, and β-tubulin. The bars represent the SEM of the data from 3 samples per group. *, P<0.05, ** P<0.01 compared to the hemin group. #, P<0.05, ##, P<0.01 compared with the Gap19+hemin group. [file 12974_2020_1978_MOESM1_ESM.tif]
